# Solution-Plasma-Mediated Synthesis of Si Nanoparticles for Anode Material of Lithium-Ion Batteries

**DOI:** 10.3390/nano8050286

**Published:** 2018-04-27

**Authors:** Genki Saito, Hitoshi Sasaki, Heishichiro Takahashi, Norihito Sakaguchi

**Affiliations:** 1 Faculty of Engineering, Hokkaido University, Kita 13 Nishi 8, Kitaku, Sapporo 060-8628, Japan; takahash@ufml.caret.hokudai.ac.jp (H.T.); sakaguchi@eng.hokudai.ac.jp (N.S.); 2 Kankyou-Engineering Co., Ltd. Kita 19 Higashi 1, Higashiku, Sapporo 065-0019, Japan; ht-sasaki@kankyou-eng.co.jp

**Keywords:** solution plasma, nanoparticles, batteries, silicon, anode materials

## Abstract

Silicon anodes have attracted considerable attention for their use in lithium-ion batteries because of their extremely high theoretical capacity; however, they are prone to extensive volume expansion during lithiation, which causes disintegration and poor cycling stability. In this article, we use two approaches to address this issue, by reducing the size of the Si particles to nanoscale and incorporating them into a carbon composite to help modulate the volume expansion problems. We improve our previous work on the solution-plasma-mediated synthesis of Si nanoparticles (NPs) by adjusting the electrolyte medium to mild buffer solutions rather than strong acids, successfully generating Si-NPs with <10 nm diameters. We then combined these Si-NPs with carbon using MgO-template-assisted sol-gel combustion synthesis, which afforded porous carbon composite materials. Among the preparations, the composite material obtained from the LiCl 0.2 M + H_3_BO_3_ 0.15 M solution-based Si-NPs exhibited a high reversible capacity of 537 mAh/g after 30 discharge/charge cycles at a current rate of 0.5 A/g. We attribute this increased reversible capacity to the decreased particle size of the Si-NPs. These results clearly show the applicability of this facile and environmentally friendly solution-plasma technique for producing Si-NPs as an anode material for lithium-ion batteries.

## 1. Introduction

Rechargeable lithium-ion batteries (LIBs) have been widely used as energy-storage devices for applications such as portable electronic devices and electric vehicles. Among the newer anode materials with higher capacities, silicon anodes have attracted considerable attention because of their high theoretical capacity of 4200 mAh·g^−1^, which exceeds that of commercialized graphite anodes [1,2,3,4]. During the lithium insertion-extraction process, however, a large volume change (>280%) inevitably occurs, which leads to pulverization of the silicon anode and loss of electrical contact with the current collector, resulting in poor cycling performance [2,5]. To mitigate this volume-change issue, several strategies have been proposed, including reducing the particle size to nanoscale [6,7], fabricating Si nanostructures such as nanowires and nanoporous materials [8,9,10,11], utilizing hollow core-shell structures [12], and dispersing nano-Si in a conductive carbon matrix to form Si-carbon composites [13,14,15,16,17,18,19]. Liu et al. clarified that the critical particle diameter for a Si anode should be less than 150 nm to avoid surface cracking and subsequent fracturing during lithiation [5]. In addition, dispersing silicon nanoparticles (Si-NPs) into a carbon matrix is a technique that has been well developed; here, the carbonaceous material acts to buffer the volume expansion and improves the electrical conductivity of the Si active materials [13].

As an effective synthetic route for Si-NPs, this study proposes the solution-plasma-mediated synthesis [20,21,22,23,24,25,26,27,28,29,30]. In this process, Si-NPs are directly synthesized from a Si bar electrode via a solution-plasma treatment. Our previous study revealed that the use of a strong acid electrolyte solution was effective for producing Si-NPs without oxidation [31]. In general, the solution plasma technique offers many advantages, such as (1) simple experimental setup, (2) use of readily available precursors, and (3) applicability to mass production. Unfortunately, with respect to the last point, strong acid solution is not applicable on large scale. Furthermore, the performance of LIBs based on Si-NPs synthesized from solution plasma is still unclear. Therefore, in this study, we have optimized the Si-NPs synthesis conditions using mild buffer solutions.

In addition to the Si-NPs synthesis, the fabrication of a composite material consisting of the Si-NPs and porous carbon is also important for overcoming the volume-change issue. This study applied a sol-gel solution-combustion synthesis (SCS) approach, which is a highly exothermic and self-sustaining process involving heating a homogeneous solution of aqueous metal salts and fuels such as urea, citric acid, glycine acid, or glycine [32,33,34,35,36,37]. This method has been applied to synthesize a Sn-NP-embedded porous carbon structure, using nanosized MgO as the template upon which to construct the porous structure; this material displayed good cycle performance as an LIB anode [38]. Based on this result, a Si-C composite material was synthesized via MgO template-assisted SCS, in which the starting material was a gel containing the Si-NPs, glycine (C_2_H_5_O_2_N) as the carbon source, and Mg(NO_3_)_2_·6H_2_O as the template. After the combustion reaction, the generated MgO was removed from the carbon, leaving the Si-NPs dispersed throughout the porous carbon structure after calcination in N_2_. The obtained materials were characterized by X-ray diffractometry (XRD) and transmission electron microscopy (TEM). Finally, the electrochemical properties of the product as an LIB anode material were investigated.

## 2. Materials and Methods

**Figure 1:**
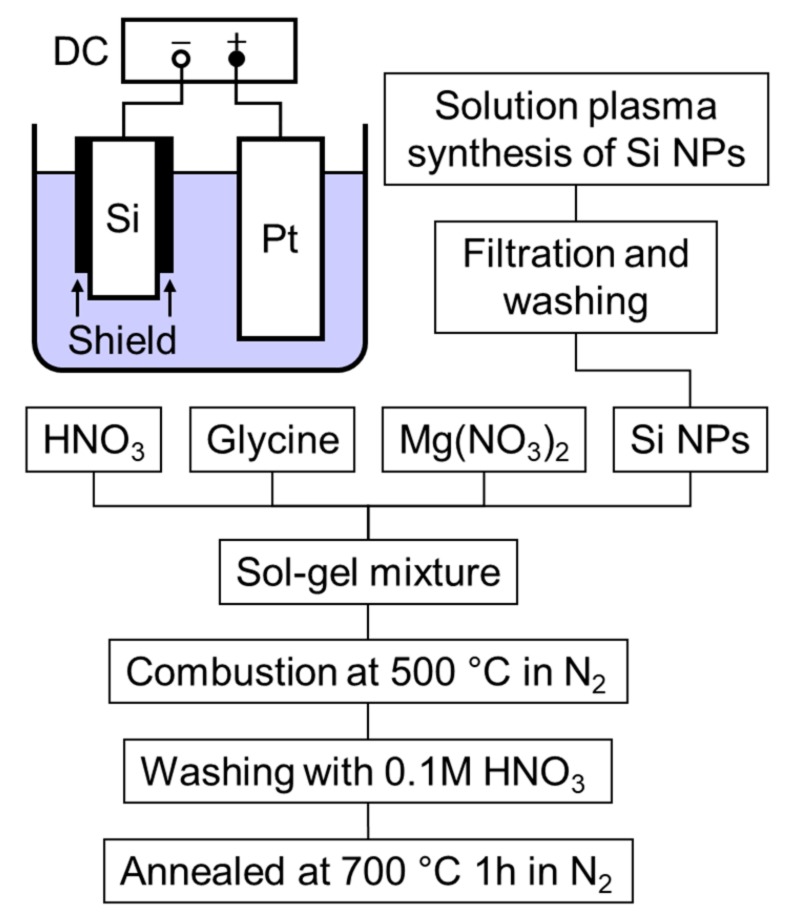
(Color online) Experimental setup for producing Si particles and schematic diagram for the solution combustion synthesis of Si-C the composite.

Figure 1 shows the experimental setup for producing the Si particles and a schematic diagram for the solution-combustion synthesis of the Si-C composite. A B-doped p-type Si bar with a square cross-sectional width of 5.0 mm (Shin-Etsu Chemical Co., Ltd., Tokyo, Japan) and electrical resistance of 0.00494–0.00478 Ω cm was used as the cathode. The upper part of the Si bar was shielded by a quartz glass tube to generate a plasma at the bottom tip of the electrode. A counter electrode was the Pt mesh. A voltage was applied using a direct current power supply. To study the effect of the electrolyte on the generation of the Si-NPs, solutions of KCl + H_3_BO_3_, KH_2_PO_4_ + K_2_HPO_4_, and LiCl + H_3_BO_3_ were used. The electrolyte concentrations and applied voltages are summarized in Table 1. After the synthesis, the products were collected by filtration and then washed several times with deionized water.

To synthesize the Si-C composite, commercially available Mg(NO_3_)_2_·6H_2_O (1.282 g, 0.005 M) and C_2_H_5_O_2_N (2.252 g, 0.03 M) were added to 20% HNO_3_ solution (20 mL). After agitation, the Si-NPs (30 mg) synthesized as described above were added to the HNO_3_ solution and then dispersed with a thin-film spin system high-speed mixer (Filmix Model 30-L, PRIMIX Corp., Osaka, Japan) at 5000 rpm for 15 min. The resulting solution was then dried on a hot plate at 90 °C. The prepared gel was transferred to a furnace for combustion synthesis. The furnace was evacuated to below 100 Pa and then nitrogen was introduced at 2 L/min. The annealing temperature was set at 500 °C. Upon reaching 200–300 °C, the gel quickly combusted, releasing a large amount of gases. After SCS, the obtained particles were washed sequentially with 0.1 M HNO_3_ solution and deionized water to remove the MgO phase. After drying, the sample was calcined at 700 °C for 1 h under N_2_ atmosphere.

The particles obtained at the end of each step were analyzed via XRD (Miniflex600, Rigaku, Tokyo, Japan), employing Cu Kα radiation (λ = 1.5418 Å). The microstructure of the samples was observed using a field-emission scanning electron microscope (SEM; JSM–7001FA, JEOL, Tokyo, Japan), in which the inner structure of the porous particle was observed by the ion-milling technique using a cross-sectional polisher (CP, IB-19510CP, JEOL, Tokyo, Japan ). TEM imaging (JEOL JEM-2010F, Tokyo, Japan) was also performed. Raman spectra of the porous carbon were acquired from a LabRam 1B Raman spectrometer (HORIBA, Kyoto, Japan).

Electrochemical characterization was performed in two-electrode Swagelok-type cells. The working electrode consisted of the active material, conductive carbon (acetylene black), and a polymer binder of sodium carboxymethyl cellulose (CMC) and poly(acrylic acid) (PAA) in a weight ratio of 75:15:5:5. The well-blended solution-based slurry was spread onto copper foil and dried at 60 °C for 3 h under vacuum. The dried electrode was punched into a 14-mm-diameter disc with a mass loading of 2–3 mg. A metallic lithium disc (15-mm diameter) was used as the counter and reference electrodes. The cells were assembled in an Ar-filled glove box (UNICO), using a solution of 1 M LiPF_6_ dissolved in ethylene carbonate (EC)/dimethyl carbonate (DMC) (1:1 *v*/*v*) as the electrolyte and a polypropylene membrane as the separator. The cells were galvanostatically cycled from 0.01 to 2.0 V versus Li/Li^+^ at 0.5 A/g in the constant current mode using a battery charge/discharge system (MSTAT4, Arbin Instruments, College station, TX, USA) at a constant temperature of 25 °C.

## 3. Results and Discussion

The Si-NPs were synthesized using solution plasma generated by contact glow discharge electrolysis (CGDE). Two electrodes consisting of a Si bar and Pt mesh were placed in a glass cell and a direct-current voltage was applied. When the voltage was increased above 1.3 V, the current increased linearly in accordance with Ohm’s law, corresponding to the occurrence of water electrolysis. Since the electrode surface of Si is smaller than the Pt mesh, the thermal loss concentrates at the Si anode-solution interface. When a higher voltage is applied, the increased current heats the solution near the Si electrode, and finally, the solution temperature surrounding Si electrode exceeds the boiling point and a gas layer consisting of steam is generated. Once the gas layer is generated, the cathode and solution are no longer in contact and the current is decreased. When the voltage is sufficiently high (>150 V) [39,40], a discharge with intense light emission begins in the gas layer. The surface of the electrode partially melts to produce nanoparticles, owing to the concentration of the current caused by the electrothermal instability [41,42]. Control parameters for size of Si-NPs are applied voltage and plasma mode. When the plasma mode is kept as a partial plasma region, the particle size decreases with an increase of applied voltage [43,44]. However, the higher applied voltage induces the transition from partial plasma to full plasma region, in which coarse particles are formed under the high excitation temperature. Thus, we have selected the appropriate voltage for each electrolyte, as shown in Table 1.

In our previous study, K_2_CO_3_, KNO_3_, KCl, HNO_3_, and HCl electrolytes were used for Si-NPs synthesis; among these, the acid solutions were effective for producing Si-NPs without oxidation [31]. When the electrolyte was either a strong acid or strong base, the pH remained constant. The pH of KNO_3_ and KCl increased during electrolysis because of the consumption of negative ions and formation of OH^−^ ions. In principle, the electrolysis of an acidic solution can be described as follows:

2H_2_O = 4H^+^ + O_2_(g) + 2e^−^ (1)

However, the reduction of Cl^−^ ions is favored over the decomposition of H_2_O:

2Cl^−^ = Cl_2_(g) + 2e^−^ (2)

Therefore, the pH in the neutral solution increases during electrolysis due to the generation of Cl_2_ gas, and oxidized Si particles are then formed to react with the OH^−^ ions, which produce Cl^−^ ions instead. At certain hot spots on the electrode surface during the plasma electrolysis, the Si^2+^ ions were generated, and they combined with OH^−^ to form SiO_2_: Si^2+^ + 2OH^−^ → Si(OH)_2_ → SiO_2_ + H_2_O. Etching of Si in KOH and NaOH was also reported by other research groups [45,46]. Thus, the pH value of the electrolyte might have affected the product phase. In the case of KNO_3_ solution as the electrolyte, the reduction of NO_3_^−^ to NO_2_^−^ may occur at the cathode electrode [47].

NO_3_^−^ + 2e^−^ + H_2_O = NO_2_^−^ + 2OH^−^ (3)

Previously, we found that the pH values of 0.1 M KCl and KNO_3_ solutions were increased from 6.13 and 6.87 to 8.71 and 9.88, respectively, during the solution-plasma synthesis of Si-NPs [44]. When the electrolyte solution becomes too alkaline, the synthesized Si-NPs tend to be oxidized. In contrast, strong acids such as HCl and HNO_3_ are effective for Si-NPs synthesis, but such strong acids are harmful and corrosive. Thus, the use of neutral solutions is attractive because of their safety and non-corrosive natures. This study investigates the non-volatile buffer solutions, KCl 0.125 M + H_3_BO_3_ 0.125 M, KH_2_PO_4_ 0.249 M + K_2_HPO_4_ 0.001 M, and LiCl 0.2 M + H_3_BO_3_ 0.15 M. In the experiments, solution plasma is successfully generated in each electrolyte. As shown in Table 1, the changes in pH are smaller compared to the cases of neutral single-electrolyte solutions, suggesting the effectiveness of the buffered systems. The initial solution temperature was over 92 °C because the pre-warming of the electrolysis was effective for initiate the plasma generation [48]. During Si-NPs production, solution temperature was controlled in the range of 94~99 °C. 

**Figure 2:**
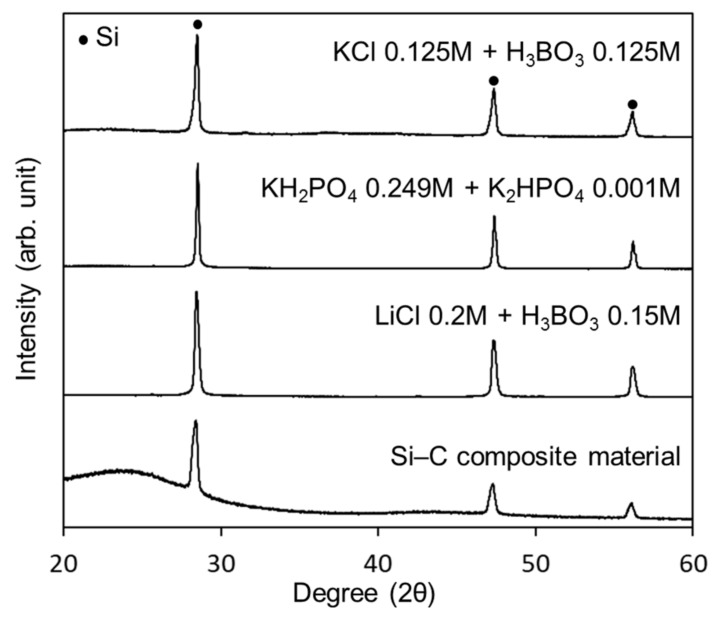
XRD patterns of the synthesized Si-NPs, as well as the Si-C composite prepared from Si-NPs generated in LiCl + H_3_BO_3_ solution.

**Figure 3:**
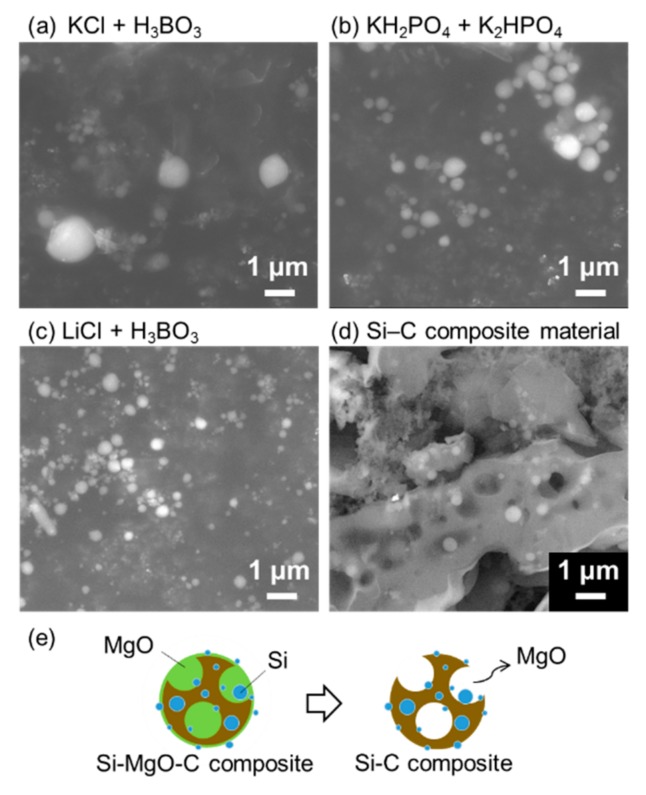
(**a**–**c**) SEM images of Si-NPs synthesized in different buffers; and (**d**) cross-sectional SEM image of Si-C composite (LiCl + H_3_BO_3_ solution); (**e**) Illustration of porous Si-C composite material.

**Figure 4:**
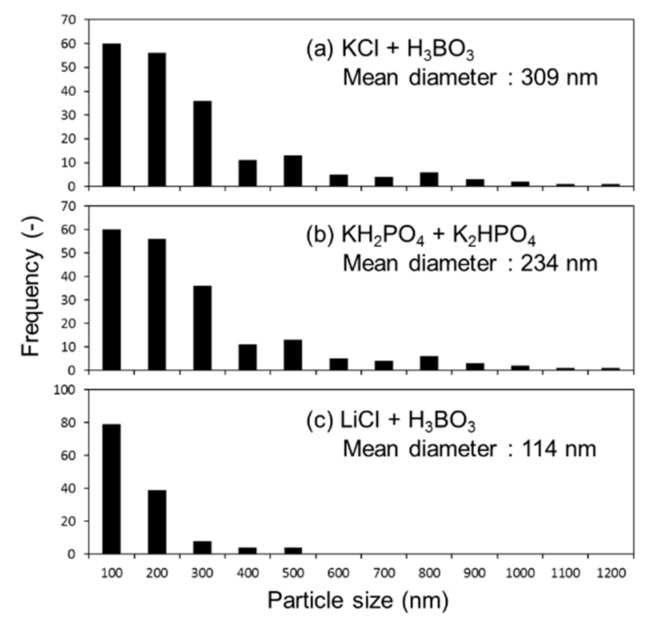
The particle size distribution of Si-NPs, in which the particle sizes with over 50 nm were analyzed using a low magnification TEM.

**Figure 5:**
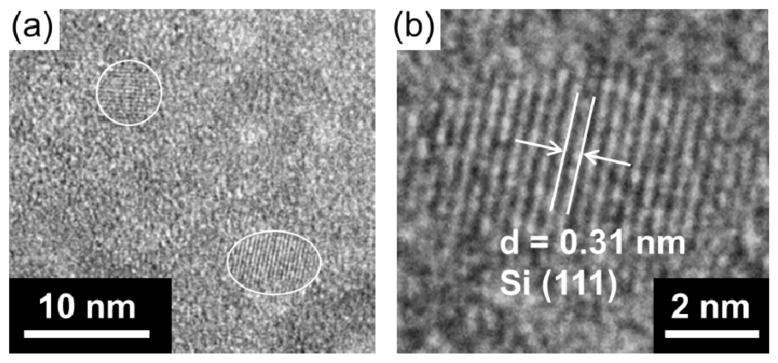
(**a**) High-resolution TEM image of Si-NPs synthesized using LiCl + H_3_BO_3_ solution. The white circle indicates the Si-NPs; (**b**) Enlarged image of Si-NPs. The obtained d spacing matched to the {111} plane of cubic diamond-structured Si.

The produced Si-NPs were collected and characterized by XRD, SEM, and TEM. Figure 2 shows the XRD patterns of the synthesized crystalline Si-NPs. generated in the three buffer solutions. Figure 3a–c shows the SEM images of the synthesized Si-NPs. The synthesized particles are almost spherical, and some particles have an oval sphere shape. During melting and solidification process, the generated particles forms sphere due to a surface tension. The particle size was measured using low magnification TEM as shown in Figure 4, in which the particle size with over 50 nm were analyzed using low magnification TEM. In the case of the KCl + H_3_BO_3_ solution, the particle size of Si particles widely distributed from 50 nm to over 1 μm. Although the KH_2_PO_4_ + K_2_HPO_4_ solution produces smaller particles, the LiCl + H_3_BO_3_ solution is the most effective for producing small Si-NPs. Although the detailed mechanism is still unclear, ions such as K^+^, Na^+^, and Li^+^ are excited in the light-emitting plasma layer [49], and these excited species might affect the generated plasma and local current concentration, resulting in the change in product size. Figure 5 shows high-resolution TEM images of the Si-NPs synthesized in the LiCl + H_3_BO_3_ solution, in which Si-NPs with diameters of less than 10 nm can also be observed. From the high-resolution TEM image shown in Figure 4b, small Si-NPs have a single-crystalline structure, in which the obtained d spacing matched to the {111} plane of cubic diamond-structured Si. 

**Figure 6:**
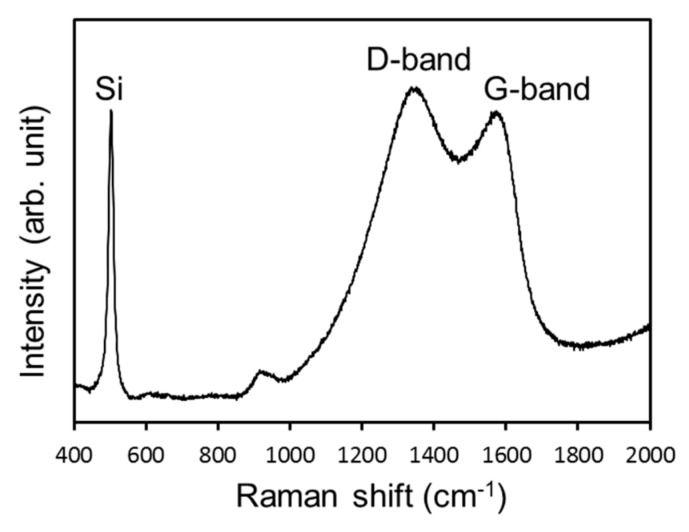
Raman spectrum of the Si-C composite prepared from Si-NPs generated in LiCl + H_3_BO_3_ solution.

For the LIB measurements, composite materials of porous carbon and Si-NPs were synthesized via MgO template-assisted SCS to accommodate the huge volume expansion of Si-NPs during the lithiation reaction. As indicated in Figure 1, the starting material is a gel containing the Si-NPs, glycine as the carbon source, and Mg(NO_3_)_2_·6H_2_O as the template. After the combustion reaction, the generated MgO is removed from the carbon, and the Si-NPs remain dispersed throughout the porous carbon structure after calcination under N_2_, as shown in the schematic illustration in Figure 3e. This process has been applied to fabricate a composite material of Sn-NPs and carbon, which successfully improved battery properties [37]. Figure 2 shows the XRD pattern of the synthesized Si-C composite. After combustion synthesis, the crystal structure of the Si is unchanged and a broad peak originating from the carbon structure has appeared. Figure 6 shows the Raman spectrum of the Si-C composite. The peak at around 500 cm^−1^ corresponds to a silicon band [50,51]. Two strong peaks at around 1350 and 1570 cm^−1^ are D-band and G-band of carbon, indicating the disordered carbon and ordered graphitic carbon, respectively. The ratios of the intensities of the D-band to G-band, *I*_D_/*I*_G_, are higher than 1 for Si-C composite. This indicates that the synthesized porous carbon has disordered carbon structure, rather than highly graphitic structure. It is reported that the generated carbon contains nitrogen after SCS using glycine and nitrate [38,52]. The existence of nitrogen might affect to the crystallinity of the generated carbon.

**Figure 7:**
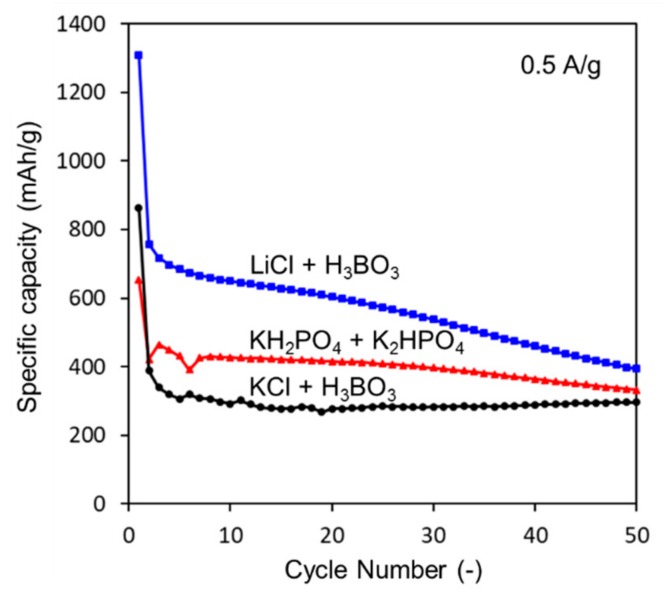
Cycling performance of Si-C composite anode materials at 0.5 A/g ranging from 0.01 to 2.00 V.

The electrochemical properties of the synthesized composites were investigated using a Li disk as the reference and counter electrodes at a current density of 0.5 A/g. Figure 7 shows the cyclic performance of the materials synthesized in the different electrolytes. The Si-C composite containing the LiCl + H_3_BO_3_ solution-based Si-NPs shows a higher initial discharge capacity (over 1300 mAh/g) compared to the other electrolyte-derived composites, and the capacity gradually decreases with increasing cycle number. This same composite material (using Si-NPs from prepared in LiCl + H_3_BO_3_ solution) exhibits a higher reversible capacity (537 mAh/g) after 30 discharge/charge cycles at a current rate of 0.5 A/g, compared to the 283 mAh/g observed for the composite prepared with KCl + H_3_BO_3_ solution-based Si-NPs. Because the theoretical capacity of graphite is 360 mAh/g, these results reveal that the synthesized Si-NPs can function as anode materials. The difference in capacity is mainly due to the particle size of the Si-NPs. When the particle size of the Si-NPs is less than 150 nm [5], their fracture during cycling can be avoided. Thus, the small Si-NPs produced in the LiCl + H_3_BO_3_ solution are effective for increasing the capacity. However, the discharge capacity continuously decreases. Because the volume expansion of the Si-NPs during lithiation exceeds 400%, the synthesized porous structure might be insufficient to accommodate this expansion, resulting in the fracture of the carbon electrode. In the case of the Sn-C composite synthesized by the solution-combustion method [38], the synthesized SnO_2_ nanoparticles are reduced by the surrounding carbon to form a Sn melt during calcination. This Sn melt moves to the surface of the porous carbon, resulting in the “attachment” of the Sn nanoparticles to the carbon structure to afford excellent battery performance. In the case of the Si-NPs, the solid Si-NPs are “embedded” in the carbon structure, as shown in Figure 3d, which might have induced the pulverization of the composite. A smaller particle size, use of a polymer, and a well-organized porous structure should increase the cycle stability.

## 4. Conclusions

In this study, Si nanoparticles (Si-NPs) were synthesized via a facile solution-plasma-mediated synthesis, and composites consisting of Si-NPs and porous carbon were fabricated as the anode materials for lithium-ion batteries. Among several mild buffer solutions investigated, the LiCl 0.2 M + H_3_BO_3_ 0.15 M solution effectively produced smaller Si-NPs. These Si-NPs were formed from a solid Si bar via local current concentration during the solution-plasma treatment. High-resolution TEM imaging confirmed the synthesis of Si-NPs with diameters of less than 10 nm. The Si-C composite material was synthesized via MgO template-assisted solution-combustion synthesis. The composite material obtained from the LiCl 0.2 M + H_3_BO_3_ 0.15 M solution exhibited a reversible capacity of 537 mAh/g after 30 discharge/charge cycles at a current rate of 0.5 A/g, as compared to the 283 mAh/g observed for the KCl 0.125 M + H_3_BO_3_ 0.125 M solution. The increased reversible capacity is mainly due to the decreased particle size of the Si-NPs. These results clearly show the applicability of this facile and environmentally friendly solution-plasma technique for producing Si-NPs as an anode material for LIBs.

## Figures and Tables

**Table 1:** Summary of experimental conditions and changes in pH and electrical conductivity for each electrolyte.

Electrolyte	Voltage (V)	Current (A)	Before			After		
			pH (-)	Electrical Conductivity (mS/m)	Electrolyte Temperature (°C)	pH (-)	Electrical Conductivity (mS/m)	Electrolyte Temperature (°C)
KCl 0.125 M + H_3_BO_3_ 0.125 M	195	2.01	4.84	1509	93.4	-	-	94.8
KH_2_PO_4_ 0.249 M + K_2_HPO_4_ 0.001 M	189	2.46	4.54	1879	92.3	4.59	1848	95.6
LiCl 0.2 M + H_3_BO_3_ 0.15 M	180	1.56	5.11	1716	94.7	7.52	1670	98.7
